# A MADS-box gene-induced early flowering pear (*Pyrus communis* L.) for accelerated pear breeding

**DOI:** 10.3389/fpls.2023.1235963

**Published:** 2023-09-25

**Authors:** Sumathi Tomes, Kularajathevan Gunaseelan, Monica Dragulescu, Yen-Yi Wang, Lindy Guo, Robert J. Schaffer, Erika Varkonyi-Gasic

**Affiliations:** ^1^ The New Zealand Institute for Plant & Food Research Ltd (PFR), Auckland, New Zealand; ^2^ The New Zealand Institute for Plant & Food Research Ltd (PFR), Motueka, New Zealand; ^3^ School of Biological Sciences, The University of Auckland, Auckland, New Zealand

**Keywords:** pear, flowering, MADS-box gene, BpMADS4, fast breeding

## Abstract

There have been a considerable number of studies that have successfully sped up the flowering cycle in woody perennial horticultural species. One particularly successful study in apple (*Malus domestica*) accelerated flowering using a silver birch (*Betula pendula*) *APETALA1/FRUITFULL* MADS-box gene *BpMADS4, w*hich yielded a good balance of vegetative growth to support subsequent flower and fruit development. In this study, *BpMADS4* was constitutively expressed in European pear (*Pyrus communis*) to establish whether this could be used as a tool in a rapid pear breeding program. Transformed pear lines flowered within 6–18 months after grafting onto a quince (*Cydonia oblonga*) rootstock. Unlike the spindly habit of early flowering apples, the early flowering pear lines displayed a normal tree-like habit. Like apple, the flower appearance was normal, and the flowers were fertile, producing fruit and seed upon pollination. Seed from these transformed lines were germinated and 50% of the progeny flowered within 3 months of sowing, demonstrating a use for these in a fast breeding program.

## 1 Introduction

Woody perennials undergo a long juvenile stage before they acquire reproductive competence (Bergonzi and Albani, 2011; van Nocker and Gardiner, 2014). Apple (*Malus domestica*) can take from 5 to 12 years before first flowering (Fischer, 1994). Similarly, European pear (*Pyrus communis*) has a long juvenile phase and can take over 10 years to first flowering (Visser, 1964; Freiman et al., 2011). The average juvenile period for European pear seedlings grown on their own roots is eight years (Brewer et al., 2008), but many pears require as much as 14 years for first flowering, or they fail to flower in research glasshouse conditions. Orchard management and agronomic practices can help reduce juvenility, with crops like apple being grafted onto a dwarfing rootstock such as ‘Malling 9’ (‘M9’) to promote faster flowering (Beakbane and Thompson, 1940). However, there are currently no pear rootstocks conferring substantial vigor control and precocity to the grafted scion cultivar (Watson et al., 2012; Knäbel et al., 2015).

Transgenic overexpression of flowering genes can promote floral induction in trees (Putterill and Varkonyi-Gasic, 2016). The molecular regulation of flowering time in plants has been very well established and is mostly evolutionary conserved, with FLOWERING LOCUS T (FT) peptides strongly promoting flowering, and highly similar but strong competitive antagonists TERMINAL FLOWER (TFL) and CENTRORADIALIS (CEN) inhibiting flowering (Wickland and Hanzawa, 2015). Overexpression or viral delivery of *FT* homologs accelerated flowering in woody perennials, including apple, poplar, citrus, plum, pear, kiwifruit, and *Eucalyptus* (Endo et al., 2005; Böhlenius et al., 2006; Hsu et al., 2006; Zhang et al., 2010; Srinivasan et al., 2012; Song et al., 2013; Wenzel et al., 2013; Yamagishi et al., 2014; Klocko et al., 2016; Voogd et al., 2017; Moss et al., 2018; Herath et al., 2023). These methods significantly reduced juvenility and induced flowering, but they have often proven to be impractical, giving rise to plants that were flowering in the tissue culture stage and commonly developing aberrant flowers. A similar effect was achieved using silencing of the *TERMINAL FLOWER1* (*TFL1*) genes in apple and pear (Kotoda et al., 2006; Freiman et al., 2011; Flachowsky et al., 2012; Yamagishi et al., 2016). Recently, gene editing of *CEN* genes gave rise to early flowering kiwifruit species (Varkonyi-Gasic et al., 2019; Herath et al., 2023), whilst mutagenesis of *CEN1* and overexpression of *FT1* or *FT2* resulted in precocious flowering in poplar (Sheng et al., 2023). Editing of *TFL1* for early flowering was also performed in apple and pear (Charrier et al., 2019); however, this approach has problems including too early (*in vitro*) flowering, complex gene editing profiles and mosaicism, low transformation efficiency for pear, and the recessive nature of the mutation.

Ectopic expression of MADS-box genes associated with early floral development was also successfully used to promote maturity in trees, while providing a good balance of vegetative growth before floral production. For example, Arabidopsis *APETALA1* (*AP1*) drastically reduced the length of the juvenile phase in a *Citrus* rootstock, which flowered within 12–18 months instead of 6–7 years (Peña et al., 2001). The transgenic adult trees displayed normal development as they retained the ability to respond to environmental cues that induce flowering every year, and flowering corresponded with the spring season. The flowers were regular and fertile, and the fast-maturity trait was inherited as dominant. Overexpression of another MADS box gene, the silver birch (*Betula pendula*) *AP1*/*FRUITFUL* (*AP1/FUL*)-like *BpMADS4* was able to induce early flowering in heterologous species (Elo et al., 2007) and is used for accelerated breeding in apple (Flachowsky et al., 2007; Flachowsky et al., 2011).

Long juvenility is a major constraint for pear breeding (Fischer, 2009). This is despite opportunities arising from the sequencing of pear genomes (Wu et al., 2013; Chagné et al., 2014; Gao et al., 2021) and targeted genome editing opportunities (Charrier et al., 2019). To establish whether we could get precocious flowering in pear, we first cloned the *BpMADS4* gene in a new transformation vector and tested the ability to promote flowering in the apple ‘Royal Gala’ cultivar. This construct was then used to generate pear lines to test whether similar precocious flowering could be achieved in pears to vastly accelerate pear breeding.

## 2 Materials and methods

### 2.1 Plant material and growth conditions

Plant material from *M. domestica* Borkh. ‘Royal Gala’ (apple) and *P. communis* L. ‘Conference’ (European pear) was used for transformation. Tissue culture plants were grown in 290 mL clear, wide-mouth plastic vessels with snap-on lids containing approximately 50 mL of media. Tissue culture plant material was maintained at 25°C and a photoperiod of 16 h light (40 μmol m^−2^ s^−1^, as provided by cool-white, fluorescent light) and 8 h dark periods. Tissue culture-grown apple and pear shoots were grafted onto ‘M9’ apple rootstock and *Cydonia oblonga* ‘Quince C’ rootstock, respectively. Grafting was performed as shown (https://youtu.be/bphgFprxtXA). Glasshouse conditions had supplementary light and heat during the winter periods and were maintained at temperature min. 18°C/max. 30°C, 14 h/10 h light/dark. Seeds were harvested from the fruit and allowed to dry for 7 days. Imbibed seeds were placed in damp vermiculite and placed at 4°C for 12 weeks until they germinated. Seedlings were then transferred to potting mix and grown in the glasshouse.

### 2.2 Construct design

The *BpMADS4* full-length coding sequence (Elo et al., 2001) (Genbank accession X99654, nucleotides 83-865) with added BamHI and XbaI restriction enzyme sites was synthesized (GenScript, https://www.genscript.com/) and cloned into pSAK778, positioning it between the CaMV *35S* promoter and *OCS* terminator. The construct identity was confirmed by sequencing, then it was transformed into *Agrobacterium tumefaciens* strains LBA4404 by electroporation. Single colonies were selected and checked for the correct plasmid by sequencing. The *Agrobacterium* culture was grown overnight (250 rpm, 28°C) in YEB liquid medium (Vervliet et al., 1975), pelleted, resuspended and adjusted to an optical density at 600 nm (OD600) of 0.6 with sterile water.

### 2.3 Plant transformation

Apple leaf sections were transformed with pSAK778 *35S:BpMADS4* using *A. tumefaciens*-mediated transformation and selected on media containing kanamycin according to the previously described protocol (Yao et al., 1995). Regenerated apple shoots were grown on selection medium before grafting onto ‘M9’ apple rootstock. Wild-type (WT) stock plants grown on media without kanamycin selection and grafted onto ‘M9’ rootstock were used as control.

To establish pear shoot cultures, shoots with single nodes were collected from glasshouse-grown plants, surface‐sterilized using sodium hypochlorite (1.5%) for 12 minutes, washed in sterile water and grown in a micropropagation medium composed of Murashige and Skoog (MS) (Murashige and Skoog, 1962) macro- and micronutrients, supplemented with additional macronutrients: MgSO_4_·7H_2_O (0.185 g l^−1^), CaCl_2_·2H_2_O (0.44 g l^−1^) and KH_2_PO_4_ (0.17 _g_ l^−1^); B5 vitamins as described by Gamborg et al. (1968); hormones N^6^-benzyladenine (BA) (1.0 mg l^−1^) and indole-3-butyric acid (IBA) (0.1 mg l^−1^); sucrose (3%), Difco agar (3 g l^-1^) and Phytagel (1.7 g l^-1^), with pH adjusted to 5.8. Fully opened young expanding leaves excised from 4- to 5-week-old shoot cultures were used as explants for genetic transformation.

For pear transformation, a protocol developed by Zhu (2000) and Zhu and Welander (2004) was used, with modifications. Regeneration media containing Quoirin & Lepoivre (QL) macronutrients modified according to Leblay et al. (1991), supplemented with Fe-Na-EDTA (40 mg l^-1^), vitamins modified from Brisset et al. (1988): myo-inositol (100 mg l^-1^), thiamine (0.8 mg l^-1^), nicotinic acid (1 mg l^-1^), pyridoxine HCI (1 mg l^-1^), glycine (4 mg l^-1^); variable MS micronutrient (Murashige and Skoog, 1962) and hormone thidiazuron (TDZ) and 1-naphthaleneacetic acid (NAA) composition (**Table 1**) were evaluated. All media for transformation contained sorbitol (40 mg l^-1^), Phytagel (2.5 g l^-1^), and were adjusted to pH 5.8 prior to autoclaving at 121°C for 20 minutes, followed by addition of filter-sterilized antibiotic solutions (where required). Components for tissue culture media were purchased from Merck (Rahway, NJ, USA) and Sigma-Aldrich (St. Louis, MO, USA).

**Table 1:** Regeneration media composition.

	Media			
Component	RE1	RE2	RE3	RE4
MS micro nutrients	½ x	½ x	1 x	1 x
TDZ	5 mg l^-1^	3 mg l^-1^	5 mg l^-1^	3 mg l^-1^
NAA	0.75 mg l^-1^	0.4 mg l^-1^	0.75 mg l^-1^	0.4 mg l^-1^

All regeneration media contained macronutrients (mg l^-1^): NH_4_NO_3_ (607), KNO_3_ (1150), Ca(NO_3_)2.4H_2_O (297), MgSO_4_.7H_2_O (185), KH_2_PO_4_ (84); Fe-Na-EDTA (40 mg l^-1^); vitamins (mg l^-1^): myo-inositol (100), thiamine (0.8), nicotinic acid (1) pyridoxine HCI (1), glycine (4); sorbitol (40 mg l^-1^), Phytagel (2.5 g l^-1^) and were adjusted to pH 5.8 prior to autoclaving at 121°C for 20 minutes. MS, Murashige and Skoog medium; TDZ, thidiazuron; NAA, 1-naphthaleneacetic acid.

For transformation, fully opened young expanding leaves were cut transversely into three fragments in a sterile Petri dish (90 × 20 mm) containing sterile water. The water was removed, and the leaf sections were inoculated with *Agrobacterium* cultures for 15 to 20 min at room temperature with gentle shaking. The inoculated leaf sections were blotted using a sterile filter paper, placed with abaxial side on the surface of the regeneration medium without antibiotics and maintained at 25°C for 5 to 7 days. After co-cultivation, the explants were washed with sterile water, blot-dried and transferred to regeneration media supplemented with kanamycin 50 mg l^-1^ and cefotaxime 350 mg l^-1^. After 4 weeks, the explants were transferred to fresh regeneration media, and subsequently subcultured every 4 weeks to fresh media with the same selection (kanamycin 50 mg l^-1^ and cefotaxime 350 mg l^-1^) until regenerated adventitious shoots were formed. The regenerated shoots were transferred to micropropagation media (as described above) supplemented with kanamycin 100 mg l^-1^ and cefotaxime 350 mg l^-1^ for micropropagation and further selection to eliminate any remaining *Agrobacterium*. After 4–6 months, the surviving adventitious shoots were grafted onto ‘Quince C’ rootstock. WT explants propagated on media with no antibiotic selection were grafted and used as controls.

### 2.4 Isolation of nucleic acids and PCR analysis

Genomic DNA was extracted from young leaves of glasshouse-grown plants using the DNeasy Plant Mini Kit (Qiagen, Hilden, Germany) following the manufacturer’s instructions. To confirm the presence of the transgene, PCR amplification was performed using the PlatinumTaq DNA Polymerase (ThermoFisher Scientific, Waltham, MA, USA) and *BpMADS4* gene‐specific oligonucleotide primers (Flachowsky et al., 2007), with an initial denaturing step at 95°C for 5 min, then 35 cycles of 95°C for 15 s, 56°C for 15 s, and 72°C for 1 min. Amplification products were separated by gel electrophoresis. Total RNA was extracted from young leaves using the Spectrum Plant Total RNA kit (Sigma-Aldrich, St. Louis, MO, USA). For cDNA synthesis, 1 µg of total RNA was reverse-transcribed in a total volume of 20 µL using the QuantiTect Reverse Transcription Kit (Qiagen, Hilden, Germany) and real-time PCR was performed using the FastStart DNA MasterPLUS SYBR Green I reaction mix on a LightCycler^®^ 1.5 instrument (Roche Diagnostics, Basel, Switzerland), with a non-template control included in each run. Amplification was carried out with an initial denaturing step at 95°C for 5 min, then 40 cycles of 95°C for 5 s, 60°C for 5 s, and 72°C for 10 s. Relative expression was calculated using the LightCycler Software version 4 (Roche Diagnostics), normalized to ACTIN (forward primer 5′-GAACTATGAGCTTCCCGATGGC-3′, reverse primer 5′-CCAGCAGCTTCCATTCCAATGAG-3′) and presented as means ± standard error of three biological replicates.

## 3 Results

### 3.1 Efficacy of the *35S:BpMADS4* construct in apple

**Figure 1:**
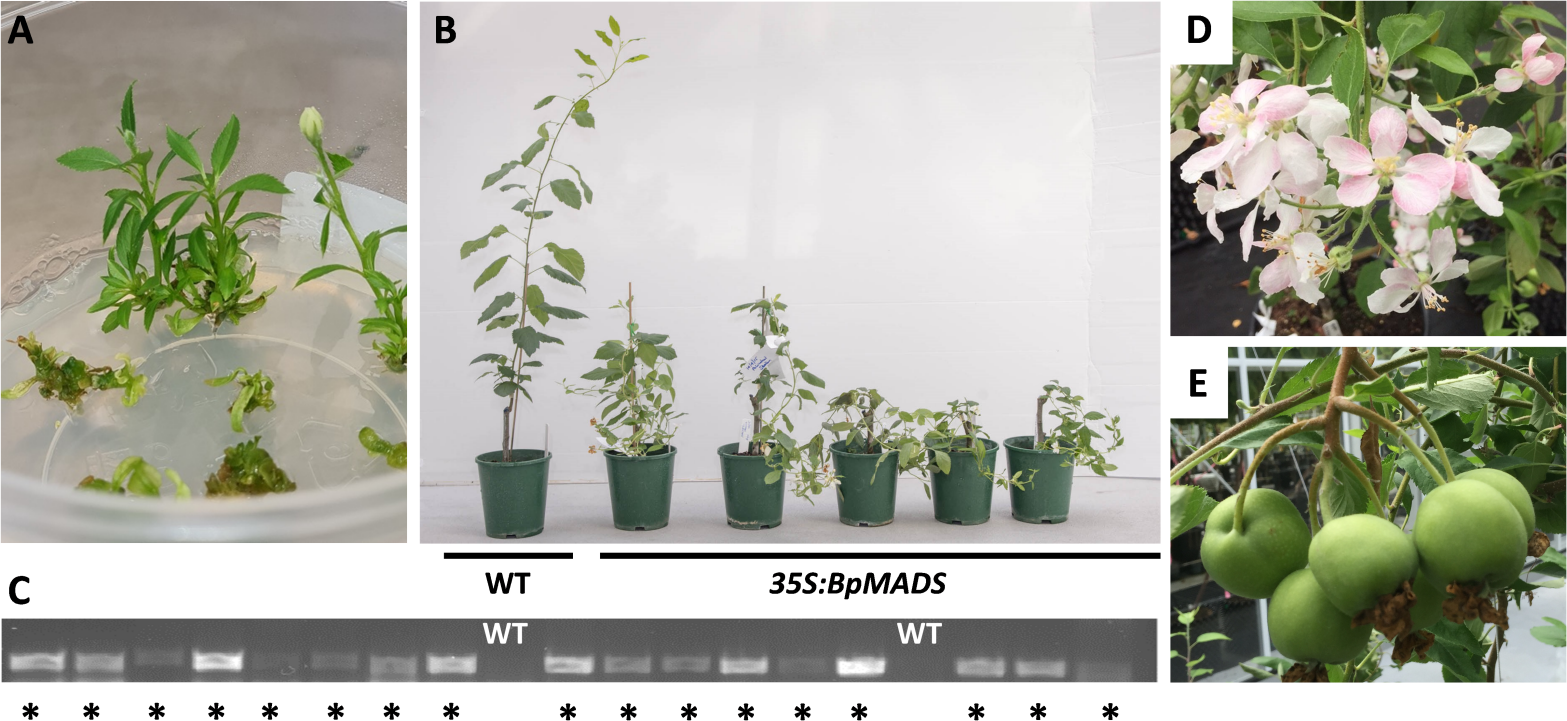
Early flowering apple. **(A)** Flower development on shoots grown *in vitro*. **(B)** Early flowering and spindly stems in *35S:BpMADS4* lines grown in the glasshouse. WT indicates the wild-type control. **(C)** Gel electrophoresis of amplified *BpMADS4* DNA fragments. Asterisks denote lines that flowered within 6 months after establishment in the glasshouse. **(D)** Normal flower development. **(E)** Fruit set after pollination.

The pSAK778 *35S:BpMADS4* construct (*35S:BpMADS4*) was used to transform ‘Royal Gala’ apple leaves. Fifty independent lines were generated and a range of precocious flowering was seen in the lines. Flowering was observed in tissue culture (**Figure 1A**) or withing weeks after grafting the shoots onto ‘M9’ rootstocks (**Figure 1B**). The presence of the *BpMADS4* transgene was confirmed in the early-flowering lines by DNA amplification compared with the WT controls (**Figure 1C**). Despite severe precocity and a spindly appearance (**Figure 1B**), most *35S:BpMADS4* lines produced flowers with normal morphology (**Figure 1D**); however, the floral clusters often contained more flowers than the typical five flower cluster observed in apple cultivars. Pollinated flowers produced apple fruit smaller than untransformed apples but typically had a similar shape to untransformed apples (**Figure 1E**). Approximately 50% of the seeds from these lines gave rise to early flowering seedlings, confirming that the *BpMADS4* transformants conferred a flowering phenotype that could be passed to the progeny.

### 3.2 Early flowering *35S:BpMADS4* pear

**Figure 2:**
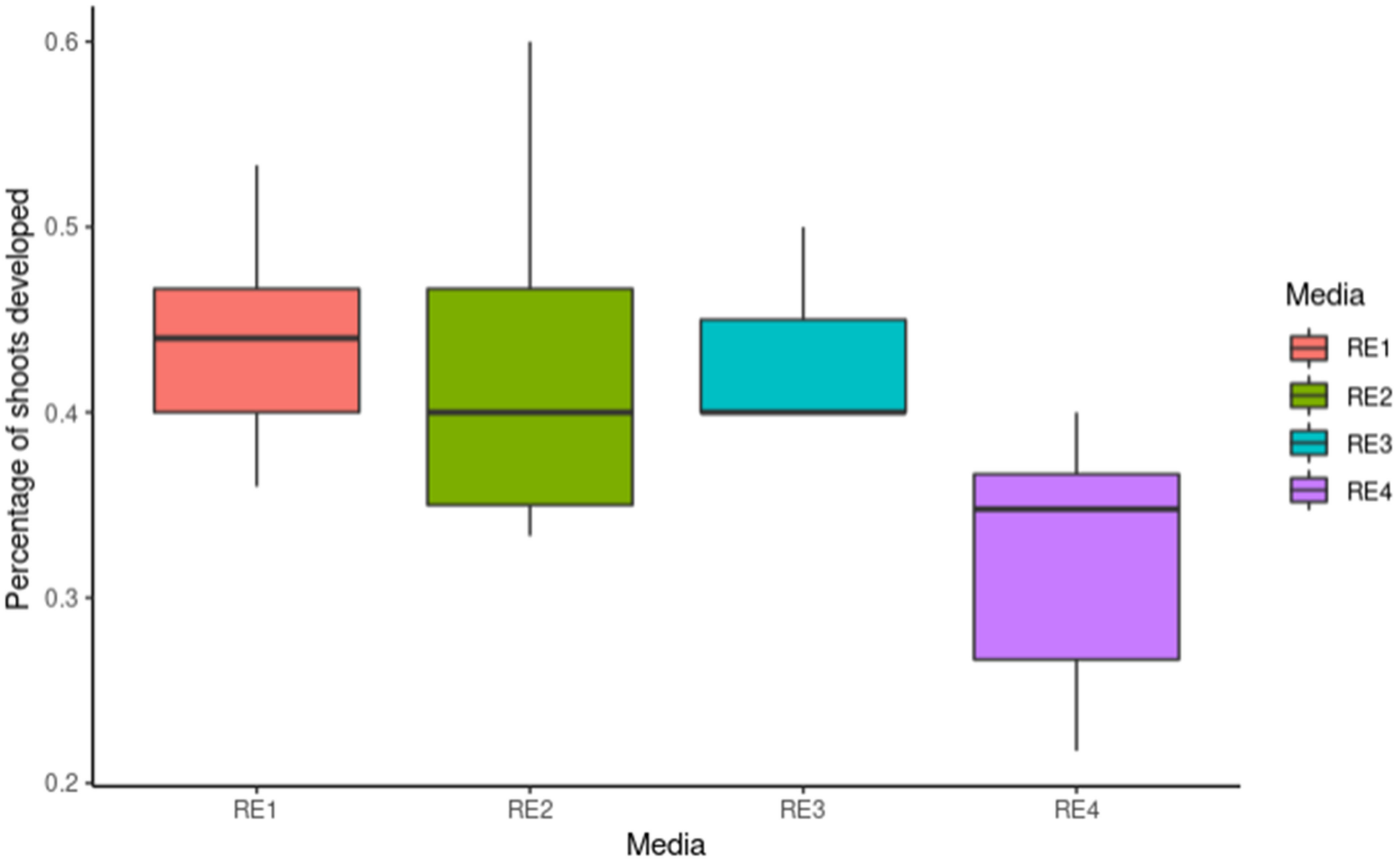
Transformation of ‘Conference’ pear. Y-axis represents frequencies of shoots regenerating from explants (n=30) propagated on different media containing kanamycin 50 mg l^-1^ for 10 weeks. The median is represented by bold horizontal line, the box indicates the interquartile range, and the minimum and maximum are shown as vertical lines.

Pear has typically been more recalcitrant to transformation than apple. Several protocols using various *Pyrus* species and cultivars, with different transformation efficiencies have been reported (Mourgues et al., 1996; Zhu, 2000; Murayama et al., 2003; Zhu and Welander, 2004; Yancheva et al., 2006; Nakajima et al., 2013; Chevreau et al., 2019). To test whether a more efficient protocol could be developed, leaf fragments from ‘Conference’ pears were inoculated with *Agrobacterium* containing *35S:BpMADS4* and placed on four different selection media (RE1, RE2, RE3 and RE4, **Table 1**), starting with no selection for 5–7 days, followed by selection on kanamycin 50 mg l^-1^ and cefotaxime 350 mg l^-1^. All media gave rise to kanamycin-resistant shoots (**Figure 2**). Roughly half of explants produced shoots on RE1, RE2 and RE3, with slightly lower regeneration efficiency noted with RE4 (**Figure 2**). However, less hyperhydricity was visually observed on tissue propagated on media RE2 and RE4, containing lower concentration of TDZ and NAA.

**Figure 3:**
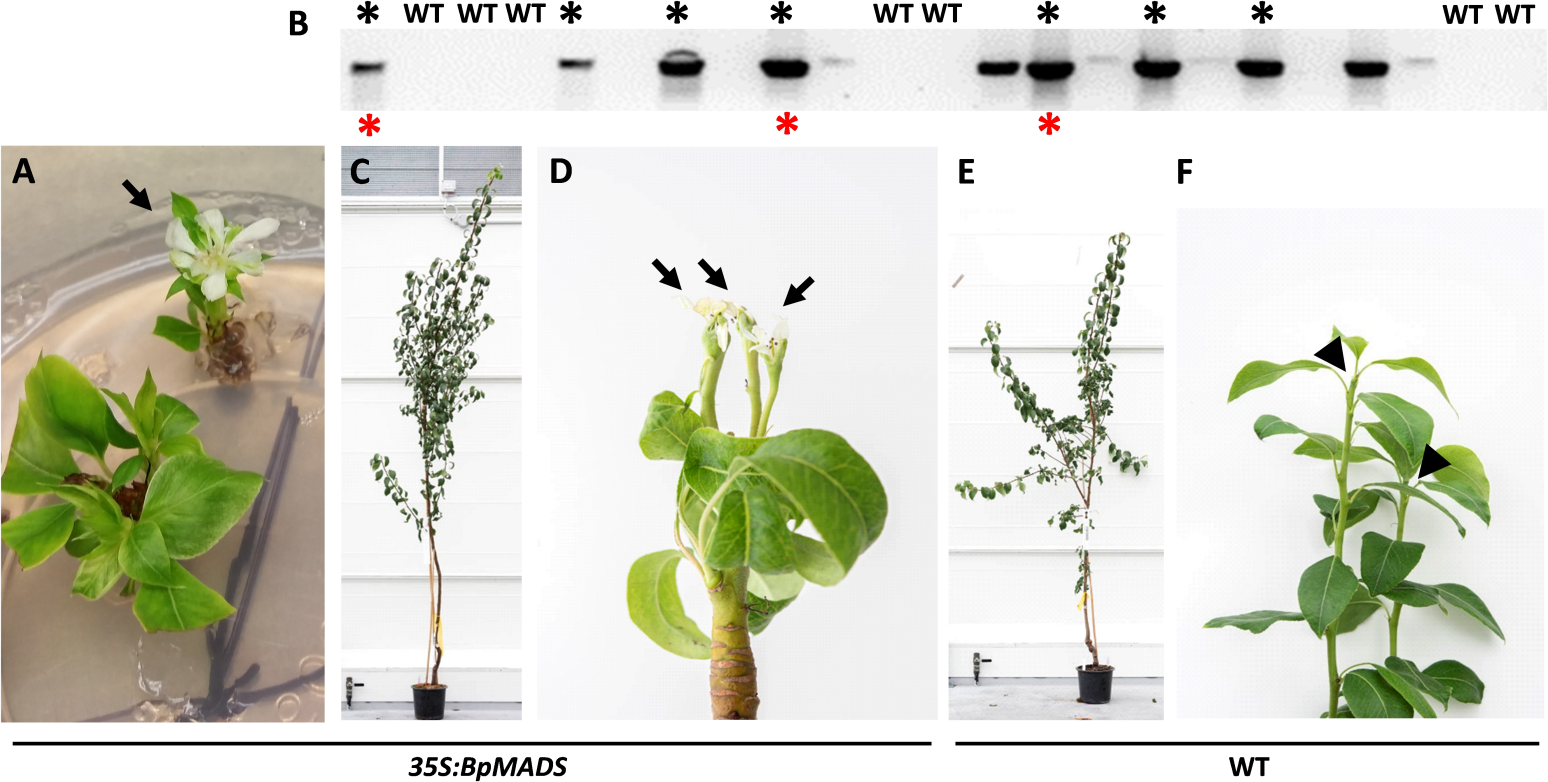
Early flowering pear. **(A)** Flower (arrow) developing *in vitro*. **(B)** Gel electrophoresis of amplified *BpMADS4* DNA fragments in kanamycin-resistant and wild-type (WT) control plants. Black asterisks denote lines that flowered 6–18 months after establishment in the glasshouse. Red asterisks indicate lines confirmed to give rise to early flowering progeny. C, **(D)** Appearance of an early flowering *35S:BpMADS4* plant **(C)** with terminal flowers (**D**, arrows). E, **(F)** Appearance of a WT control **(E)** with vegetative shoot tips (**F**, arrowheads).

**Figure 4:**
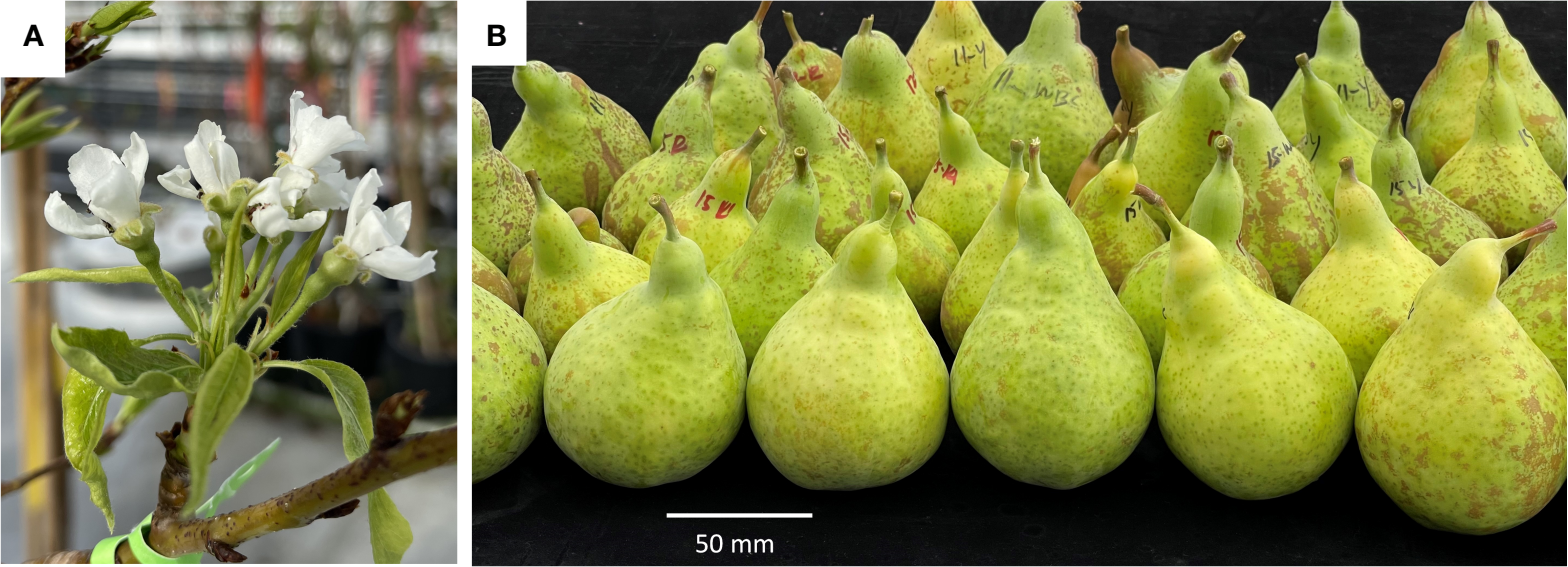
Reproductive development in the early flowering pear. **(A)** Normal flower morphology on a representative early flowering pear line. **(B)** Appearance of fruit collected from early flowering lines.

The observed phenotypes in pear shoots grown *in vitro* and in the glasshouse are shown in **Figure 3**. Occasional flowering was observed on some of the shoots developing *in vitro*, with flower and leaf morphology ranging from aberrant to mostly normal (**Figure 3A**). None of the lines demonstrating *in vitro* flowering survived. Sixteen independent lines that did not flower in tissue culture and seven controls were grafted onto quince rootstock and grown in the glasshouse. The presence of the *BpMADS4* transgene was confirmed by DNA amplification compared to the WT controls (**Figure 3B**). Of the 16 transgenic lines, seven started flowering within 6–18 months, while none of the controls flowered within this time (**Figures 3B–F**). All the transgenic lines appeared normal, with upright stems and branches and were comparable to WT control (**Figures 3C, E**). The early flowering lines had a normal flower morphology and were producing an expected cluster of five flowers per inflorescence (**Figure 4A**). The flowers were pollinated and produced medium-sized pears (150–230 g) with expected elongated shape (**Figure 4B**).

**Figure 5:**
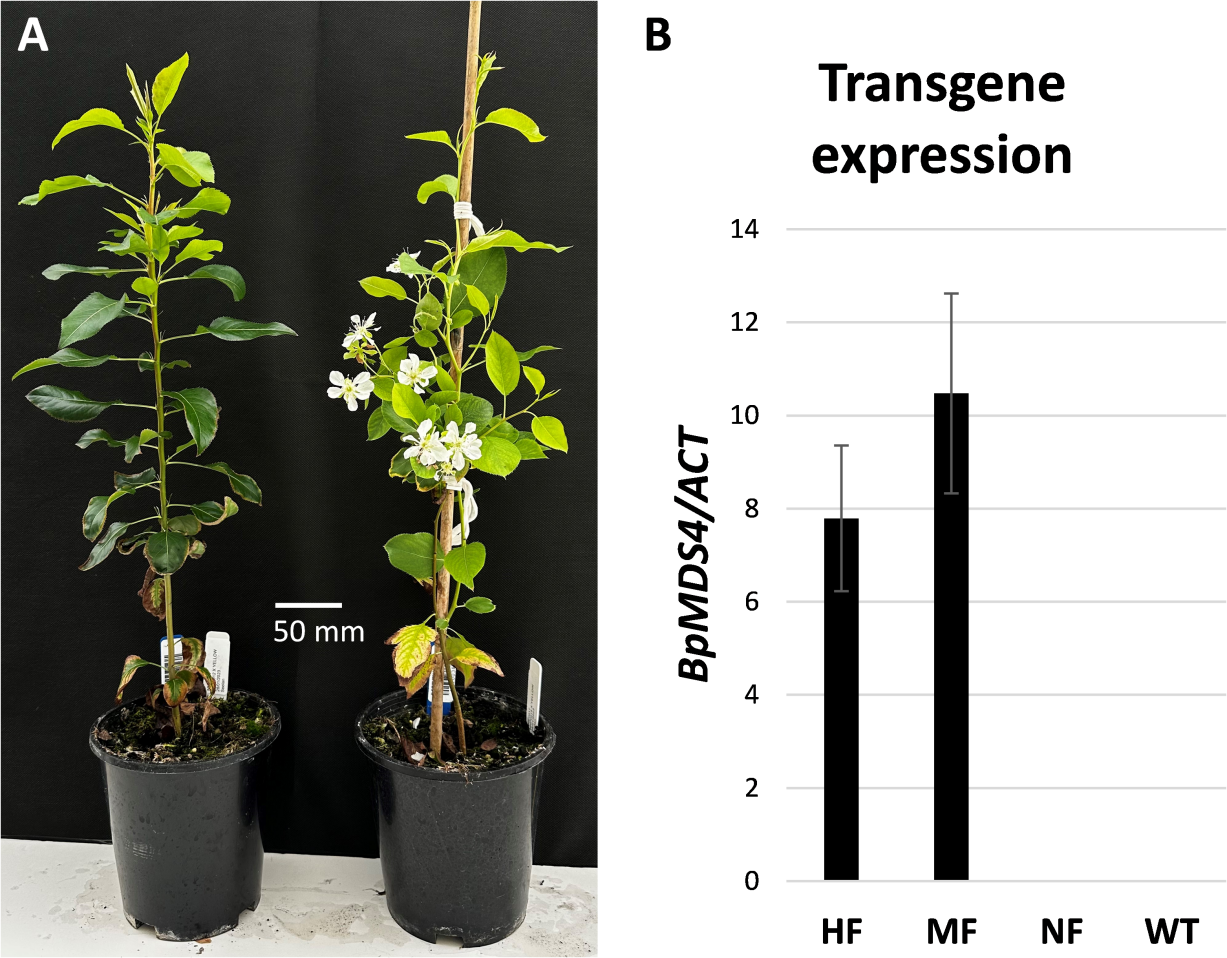
Appearance of first-generation hybrids. **(A)** Non-early flowering (left) and early flowering (right) seedlings 4 months after germination. **(B)** Relative *BpMADS4* expression normalized to *ACTIN* (*ACT*) and presented as mean ± SE of three biological replicates in early flowering highly floral (HF), moderately floral (MF) and non-flowering (NF) pear seedlings. WT, wild type.

To test whether the transgene conferred early flowering in the first-generation hybrids, a total of 48 seeds were collected from three lines (**Figure 3B**) and placed into moist conditions to germinate. A total of 40 seedlings were germinated and 15 of those seedlings flowered early, producing a terminal flower cluster 6–8 weeks after transfer to soil (**Figure 5A**). Expression of the transgene was detected in early flowering progeny (**Figure 5B**; **Supplementary Figure 1**).

## 4 Discussion

The pear industry is facing increasing obstacles such as global warming, changes in water availability, pathogens and diseases. To overcome these obstacles, traditional breeding techniques are being used to develop new and improved pear varieties. However, the slow maturation of pear seedlings is a major hurdle to conventional breeding. To produce new varieties that are resistant to pests, it is often necessary to cross to less domesticated species, resulting in multigenerational crosses needed to re-establish quality eating pears. Due to this and the long breeding cycles, conventional breeding can take several decades to produce a new pear variety. In this study, we have shown that the silver birch MADS-box gene *BpMADS4* can be used to reduce the breeding cycles to less than 2 years, greatly speeding up pear breeding. This new tool will allow wider crosses to be made, and faster introgression of desirable traits into elite lines.

Pear transformation is more challenging than other fruit crops such as apple, because of factors such as low regeneration capacity, low transformation efficiency, and genotype-dependent responses. In the majority of protocols, pear transformation is carried out using *A. tumefaciens* strains EHA101 or EHA105 and several different pear genotypes have been transformed, with relatively high transformation efficiencies reported for ‘Conference’ (Mourgues et al., 1996; Yancheva et al., 2006). An increase in transformation efficiency was reported for Agroinfiltration (*Agrobacterium*-mediated vacuum infiltration) of the explants (Chevreau et al., 2019). Here, we performed transformation using *A. tumefaciens* strain LBA4404 and evaluated four growth media for the pear explants. We found that decreased cytokinin and auxin may reduce the occurrence of hyperhydric (glassy) shoots, whilst gradually increasing kanamycin was able to improve transgenic shoot survival. Further optimization and testing of protocols may boost this efficiency further. However, propagation on all tested media resulted in ‘Conference’ transformation efficiencies comparable to those reported previously (Mourgues et al., 1996; Yancheva et al., 2006).

**Figure 6:**
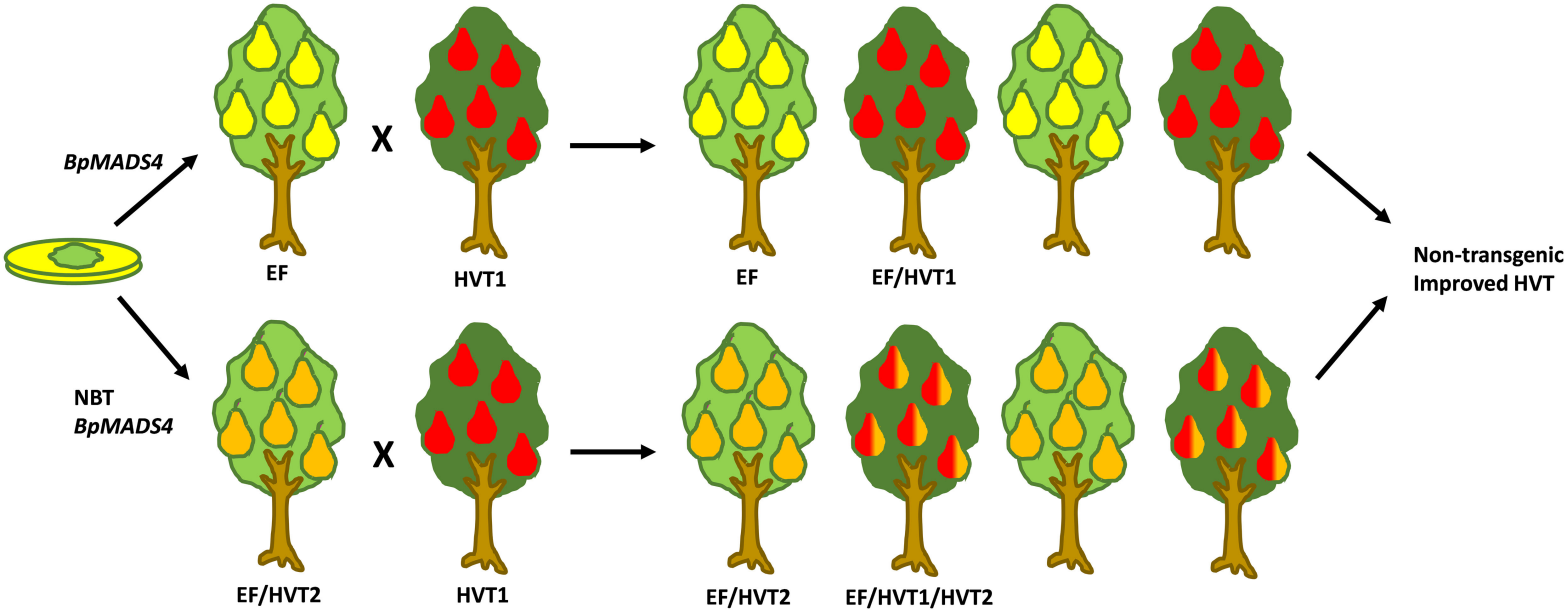
A schematic diagram representing the strategies for fast breeding and improvement of pear using early flowering *BpMADS4* lines (EF). Top, Fast breeding cycles to introduce a high value trait (HVT). Half of the progeny is transgenic (EF), with half of those inheriting the HVT (EF/HVT). Alternatively, HVTs are introduced by new breeding technologies (NBT), e.g. gene editing (bottom). After a desired number of breeding cycles, non-transgenic (not EF) improved HVT lines are selected. Different fruit colors indicate HVTs.

The early flowering observed in pear was generally later than that of the ‘Royal Gala’ transformants, with most apple lines flowering within 6 months of grafting and pear ranging from 6–18 months from grafting. Given that the pears showed some flowering in tissue culture similar to apple, we assume that the ‘M9’ rootstock used in apple assists the rapid flowering, compared with the quince rootstock. This is further supported by the seedlings from the rapid flowering pears flowering at an earlier developmental period than the pear grafted transgenics. Interestingly, the pear growth habit was less affected by the *BpMADS4* transgene than that of apple, with primary transgenic pear showing an upright, tree-like habit. The early flowering pear seedlings were also more robust than the apples, suggesting that the spindly vine morphology reported in apple containing *BpMADS4* had a different interaction with plant development processes. Expression of the transgene was detected in the early flowering progeny, but no clear correlation could be established between transgene expression and flower numbers or plant stature, demonstrating that the presence of *BpMADS4* transcript rather than small differences in its level of transcription promote the development of flowers. This was consistent with previous findings in apple (Flachowsky et al., 2007).Overall, the development of a transgenic pear with a heritable early flowering trait has the potential to significantly reduce the duration of the breeding cycle and accelerate the development of new pear varieties (**Figure 6**). Furthermore, the early flowering lines may be developed as a model pear for fruit research, opening avenues for the application of new breeding techniques such as CRISPR/Cas-mediated gene editing for further improvement of traits of interest (**Figure 6**). Finally, they can be utilized for introgression of the early flowering trait into other *Pyrus* spp. such as Asian pear (*Pyrus pyrifolia*), which is difficult to transform.

## Data availability statement

The raw data supporting the conclusions of this article will be made available by the authors, without undue reservation.

## Author contributions

EV-G and RS conceived the study, led the research and wrote the manuscript. Y-YW and EV-G designed the constructs and Y-YW performed the cloning. ST developed the tissue culture protocols, generated transgenic plants and contributed to writing of the manuscript. KG performed the genotyping, pollinated the transgenic plants and maintained the seedlings. MD performed the grafting. LG performed the statistical analysis. All authors contributed to the article and approved the submitted version.

## Supplementary material

Supplementary Video Showing pipfruit micrografting can be accessed using the following link: https://youtu.be/bphgFprxtXA. Grafting was performed by Monica Dragulescu. The video was prepared by Ben Lawrence, Donna Gibson and Tracey Phelps at The New Zealand Institute for Plant and Food Research Limited, Auckland, New Zealand.
