# Supplementary figures and images for: A MADS-box gene-induced early flowering pear (Pyrus communis L.) for accelerated pear breeding
Source: Front Plant Sci. 2023 Sep 25;14:1235963. doi: 10.3389/fpls.2023.1235963 (PMC10560987; doi:10.3389/fpls.2023.1235963)

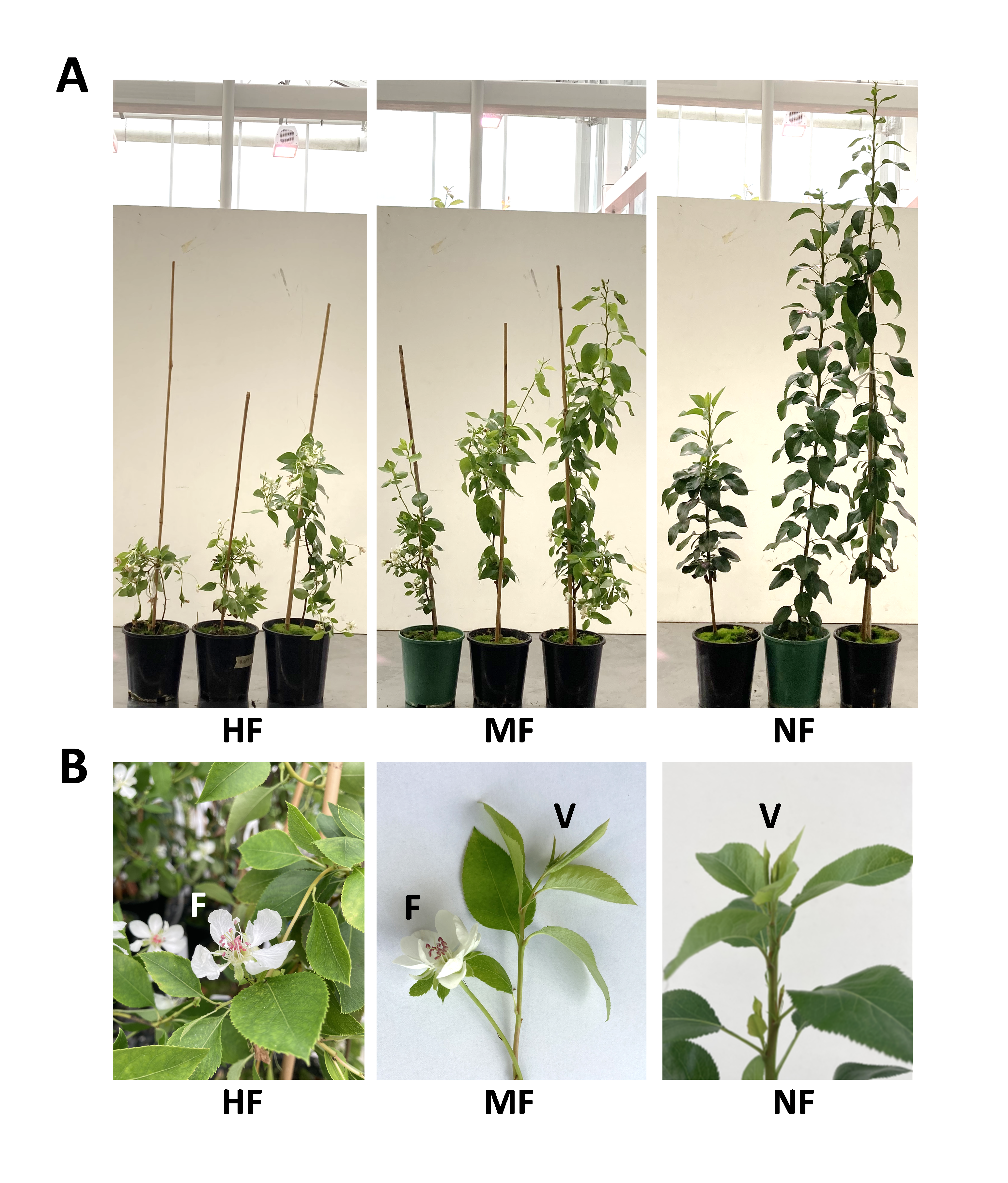

Supplement: Supplementary Video — Showing pipfruit micrografting can be accessed using the following link: https://youtu.be/bphgFprxtXA. Grafting was performed by Monica Dragulescu. The video was prepared by Ben Lawrence, Donna Gibson and Tracey Phelps at The New Zealand Institute for Plant and Food Research Limited, Auckland, New Zealand. [file Image_1.tif]
